# Hydroxyapatite Thin Films of Marine Origin as Sustainable Candidates for Dental Implants

**DOI:** 10.3390/pharmaceutics15041294

**Published:** 2023-04-20

**Authors:** Gabriela Dorcioman, Valentina Grumezescu, George E. Stan, Mariana Carmen Chifiriuc, Gratiela Pircalabioru Gradisteanu, Florin Miculescu, Elena Matei, Gianina Popescu-Pelin, Irina Zgura, Valentin Craciun, Faik Nüzhet Oktar, Liviu Duta

**Affiliations:** 1 Lasers Department, National Institute for Lasers, Plasma and Radiation Physics, 077125 Magurele, Romania; 2 National Institute of Materials Physics, 077125 Magurele, Romania; 3 Department of Microbiology, Faculty of Biology, University of Bucharest, 060101 Bucharest, Romania; 4 Earth, Environmental and Life Sciences Division, Research Institute of the University of Bucharest (ICUB), 060101 Bucharest, Romania; 5 Romanian Academy, 010071 Bucharest, Romania; 6 Academy of Romanian Scientists, 051157 Bucharest, Romania; 7 Faculty of Materials Science and Engineering, Politehnica University of Bucharest, 060042 Bucharest, Romania; 8 Department of Bioengineering, Faculty of Engineering, University of Marmara, 34722 Istanbul, Turkey; 9 Advanced Nanomaterials Research Laboratory (ANRL), University of Marmara, 34722 Istanbul, Turkey

**Keywords:** marine-derived hydroxyapatite thin film, biomaterial, cytocompatibility, antimicrobial activity, dental implant, pulsed laser deposition (PLD)

## Abstract

Novel biomaterials with promising bone regeneration potential, derived from rich, renewable, and cheap sources, are reported. Thus, thin films were synthesized from marine-derived (i.e., from fish bones and seashells) hydroxyapatite (MdHA) by pulsed laser deposition (PLD) technique. Besides the physical–chemical and mechanical investigations, the deposited thin films were also evaluated in vitro using dedicated cytocompatibility and antimicrobial assays. The morphological examination of MdHA films revealed the fabrication of rough surfaces, which were shown to favor good cell adhesion, and furthermore could foster the in-situ anchorage of implants. The strong hydrophilic behavior of the thin films was evidenced by contact angle (CA) measurements, with values in the range of 15–18°. The inferred bonding strength adherence values were superior (i.e., ~49 MPa) to the threshold established by ISO regulation for high-load implant coatings. After immersion in biological fluids, the growth of an apatite-based layer was noted, which indicated the good mineralization capacity of the MdHA films. All PLD films exhibited low cytotoxicity on osteoblast, fibroblast, and epithelial cells. Moreover, a persistent protective effect against bacterial and fungal colonization (i.e., 1- to 3-log reduction of *E. coli*, *E. faecalis,* and *C. albicans* growth) was demonstrated after 48 h of incubation, with respect to the Ti control. The good cytocompatibility and effective antimicrobial activity, along with the reduced fabrication costs from sustainable sources (available in large quantities), should, therefore, recommend the MdHA materials proposed herein as innovative and viable solutions for the development of novel coatings for metallic dental implants.

## 1. Introduction

The increase in life expectancy and the continuously ascending prevalence of bone injuries and diseases represent decisive triggers of the growing interest in devices dedicated to the dentistry and orthopedic domains. It is worth mentioning that the global implant market was evaluated in 2015 at USD 72,265 million, and is estimated to reach USD 116,300 million by 2022 [1].

Currently, implants made of titanium (Ti) or its alloys are most often used in the medical field due to some important properties, such as high corrosion resistance, low modulus of elasticity (similar to that of the bone tissue), and the ability to naturally form a thin oxide layer on the surface, which is very stable and makes them bioinert [2]. Although they possess remarkable mechanical properties, in order to boost both the osseointegration rate and biological performance of implants, their surfaces can be functionalized with thin layers of bioactive materials [3,4,5]. These coatings must fulfill some key roles: (i) to be porous and to possess hydrophilic behaviors, which involves the ability to retain water and the body’s essential fluids; (ii) to be capable of a balanced transfer of mechanical loads with low wear and/or friction values, and (iii) to facilitate the adhesion of proteins, along with the proliferation and differentiation of cells.

Thus, among the bioactive materials, the most widely applied ones are bioceramics, i.e., calcium phosphates (CaP), and bioglasses [6,7,8,9]. These materials are used mostly in medicine as coatings for various metallic implants [10], particularly in orthopedics and dentistry [11,12]. Among CaPs, great attention has been paid to hydroxyapatite (HA), Ca_10_(PO_4_)_6_(OH)_2_ [8,9,10]. Due to its appealing biological characteristics and similarity with bone mineral constituents, HA is presently one of the most widely exploited CaPs for bone substitutes in bone grafting and dental devices. It should be emphasized that the chemical composition of HA is close to that pertaining to enamel and dentin. Therefore, it was demonstrated that under physiological conditions, the HA crystals were able to induce a gradual bond with the dentine and enamel as a result of the bio-reabsorption mechanisms [13,14]. Moreover, HA-based materials have been used in toothpaste fabrication to mimic the surface reactivity, along with the compositional and morpho-structural characteristics of the pristine enamel, in order to assure home oral hygiene by efficiently sealing and promoting the remineralization of teeth surfaces [15,16,17]. The functionalization of metallic implants with thin layers of HA combines the exceptional bioactivity of the ceramic with the excellent mechanical properties of the metallic substrate [3,4,5]. HA can be of synthetic origin or obtained from natural resources [18]. The inorganic components of skeletal systems primarily consist of a non-stoichiometric and Ca-deficient composite material, with a low crystalline degree. Studies carried out on synthetic HA highlighted that it does not fully reproduce the chemical composition of the mineral phase of the human bone, because of the absence of both trace elements and functional groups [19]. However, this disadvantage was overcome by finding alternative solutions, namely, HA obtained from sustainable biological sources, such as the bones of mammals or fish [20], or biogenic materials (eggshells [21], clam shells [22]), usually considered as by-products of the food industry [23]. It has been demonstrated that CaPs extracted from biogenic sources, characterized by non-stoichiometry and a disordered structure [24], showed superior compatibility [25] and metabolic activity compared to stoichiometric materials [26]. Therefore, regarding the difference between synthetic materials and biological HA ones, the latter are much more suitable to contribute to the regeneration process of the human bone system.

Mammal bones represent a considerable source of calcium and phosphorus, as well as of trace elements [27,28,29] (the most abundant ones being Na^+^ and Mg^2+^ [30]). The presence of these ions in the chemical composition of HA materials is of key importance for the bones’ architecture, while their deficiency could have effects on bone fragility or even cause bone loss [12].

Fish bones are rich in calcium, phosphate, and carbonate, and can be considered readily available resources to produce HA (i.e., marine-derived HA, MdHA). Moreover, the HA obtained from cattle bones, in comparison to that derived from fish, is much more stable at elevated temperatures (i.e., ~1200 °C) [11].

It is important to point out that the available mineral resources are currently in danger of being exhausted due to rapid demographic growth and economic development. Thus, approaches leading towards the use of renewable sources are increasingly encouraged [31]. For example, it has been estimated that of a total of 90 million tons of fish caught globally for consumption, almost half are by-products from the processing industry [32]. Defective management of these bio-wastes, which can end up in the marine environment, can affect aquatic flora and fauna and even favor the development of parasites [33].

Besides the excellent mechanical characteristics and rapid tissue regeneration, current dental implant coatings demand the ability to create resistant seals with both connective and epithelial tissues, and to offer high antimicrobial efficiency to prevent the occurrence of infections. These infections are caused by the appearance of microbial biofilms on the surface of the implant, and are responsible for the exacerbated or prolonged inflammation of the surrounding tissue, which can ultimately lead to the removal of the implant. Therefore, the real challenge for researchers is to manufacture implantable medical devices covered with functionalized biomaterials, which combine the regenerative properties with the antimicrobial and anti-inflammatory ones. Thus, a model biomaterial must present anti-infectious characteristics, possessing physical and chemical properties that hinder early bacterial adhesion to its surface, or it must perform by delivering antimicrobial agents that are able to destroy the bacterial cells from the surroundings before reaching the surface. In general, the main pathogens involved include, but are not limited to, Gram-positive bacteria (i.e., *Staphylococcus aureus*) and fungal strains (i.e., *Candida albicans*). The reports of the National Institute of Health demonstrated that ~80% of the infections encountered in humans occur because of the formation of these biofilms [34].

It has been reported that doping of HA with various additives enhances the physical–chemical properties and shows therapeutic effects [10,33,35,36]. For example, the MgF_2_ dopant contributes to the improvement of mechanical behavior [37]. Moreover, Mg has an active role in bone metabolism. It improves the formation of new bone tissue and its mineralization by (i) stimulation of the osteoblasts’ proliferation and (ii) protection of the bone against resorption by inhibiting the osteoclasts’ activity [38]. The introduction of lithium (Li) into the HA structure can determine a solubility decline [39] without changing the biocompatibility [40], as well as an increase in the mechanical properties without changing the HA structure. The addition of 2 wt.% of MgF_2_ in biological HA demonstrated both the improvement of the adhesion of the deposited films and an increase in the antibiofilm properties [37]. It has been shown that a solution to enhance the antibacterial efficiency of HA is the introduction of small amounts of Ag (up to 2–3 wt.%) into its matrix [41]. Moreover, it should be emphasized that HA doping with Ag and Li at concentrations situated under the known limits of toxicity (i.e., up to 0.6 and 1 wt.%) was demonstrated to be unharmful to living organisms [42,43].

Among all physical vapor deposition (PVD) methods, pulsed laser deposition (PLD) is one of the most applied and effective techniques to grow thin films from a wide range of materials on substrates with complex geometries, such as implants [44]. In the case of CaPs of biological origin, the PLD technique proved to be one of the best solutions to synthesize and to transfer them, stoichiometrically, in the form of a thin layer (coating), independent of their structural and compositional complexity. This is possible due to the high ablation rate that forces all elements to evaporate simultaneously [45]. Moreover, PLD technique is recognized for its ability to stoichiometrically transfer molecules with very complex structures [46]. Another advantage of this method is that it allows for the synthesis of structures with a good control over adhesion due to the high speed of the ablated species [46,47].

Taking into consideration all of these aspects, we report in the present study a comparison, from morpho-structural, bonding strength, and biological points of view, between simple and doped (with 0.5 wt.% of Li_3_PO_4_, MgF_2_, or Ag) MdHA thin films synthesized using the PLD technique. To the best of our knowledge, this is the first comprehensive report on this combination of materials synthesized by PLD. It should be emphasized that these preliminary results show great promise for the fabrication of alternative, feasible solutions to synthetic HA for the development of a novel generation of dental implants with improved functionality.

## 2. Materials and Methods

The protocols used to fabricate MdHA materials were in agreement with EU Regulation 722/2012—New European Union animal tissue regulations in effect for some medical devices/ISO 22442-1/2015—Medical devices utilizing animal tissues and their derivatives—Part 1: Application of risk management.

### 2.1. Powders Preparation

#### 2.1.1. The Obtaining of HA from Fish Bones

Firstly, the fish (i.e., *Salmo salar*), bought from local fisheries in Turkey, were carefully cleaned of any remaining flesh parts or soft tissues to gently extract the bones. Then, the collected bony parts were boiled twice in distilled water (2 h for each operation). These were dried in a desiccator for 48 h and thermally treated (i.e., 500 °C) in an electric furnace (heating rate of 10 °C/min, for 2 h, in air with constant ventilation). To assure the full removal of all organic remnants, the fish bones were calcined in a conventional electric furnace at ~1200 °C, which ensured that no possible pathogens could remain infectious. The thermal treatments were performed for 6 h in air (heating rate of 5 °C/min). Finally, the fish bones were naturally cooled down in the furnace. The resulting bone specimens, with a white color, were next crushed into large-grained powders and further ground into fine powders for 4 h using a mill with agate balls.

#### 2.1.2. The Obtaining of HA from Seashells

The seashells (i.e., *Mytilus galloprovincialis*), harvested from the Black Sea in Romania, were first carefully washed and scrubbed for the total removal of sand particles. Afterward, they were heated in an oven to 1300 °C and maintained at this temperature for 6 h for the CaCO_3_ dissociation in calcium oxide (CaO). Next, the as-obtained powder was further hydrated with distilled water, filtered, stored in thin layer on watch glass, and dried for six days at room temperature (RT). Calcium hydroxide (Ca(OH)_2_) powder was obtained, with no residual water [48]. Then, the Ca(OH)_2_ powder was weighted using a calibrated four-decimal analytical balance (Kern & Sohn GmbH, Balingen, Germany). Additionally, the powder was treated with phosphoric acid (H_3_PO_4_, Sigma-Aldrich, St. Louis, MO, USA), in various amounts. Based on stoichiometrically-calculated amounts, 10 g of Ca(OH)_2_ was mixed with 200 mL of distilled water and 5.5 mL of H_3_PO_4_, drop-wise added at a rate of 1 mL/min, at RT. Furthermore, the resulting slurries were stirred for 2 h at 25 °C, aged for 72 h at RT, and finally dried for 2 h at 100 °C. To prevent humidification, the as-obtained powders were deposited in Petri dishes and stored in a desiccator. Next, the resulting powders were ground in a planetary mill with agate balls, then granulometrically sorted with standardized sieves (<100 μm particle size).

### 2.2. Targets Preparation

The ground MdHA powders were next doped with 0.5 wt.% of Li_3_PO_4_, MgF_2_, and Ag. The mixed powders were pressed at ~7 MPa for 2 min in a 20 mm diameter mold. The resulting cylindrical pellets (5 mm thickness) were sintered in an oven, in air, at 500 °C for 6 h. The as-obtained hard pellets were used as targets in the PLD experiments.

For an easy identification, in Table 1, the codes for all samples mentioned in the manuscript are summarized.

### 2.3. PLD Experiment

Unlike wet synthesis techniques, plasma-assisted methods offer numerous advantages, as follows: (i) a superior deposition process, (ii) stoichiometric removal of the material from the target in the deposited films, (iii) improved morpho-compositional uniformity, (iv) a low degree of porosity, (v) a reduced disposition of the synthesized films to crack or delaminate, (vi) operational safety, and (vii) low production costs. In the biomedical domain, for the manufacturing of CaP thin films for bone implant applications, one of the most used techniques is pulsed laser deposition (PLD) [30,31,32,33]. Due to the possibility of independent variation of a wide number of parameters (e.g., wavelength, laser fluence, pulse repetition rate, beam energy, target–substrate separation distance, gaseous atmosphere and substrate temperature), PLD is a versatile technique used to obtain thin layers with a great diversity of morphological, compositional and structural characteristics.

For the PLD experiments, a KrF* excimer laser source, model COMPex Pro205 (λ = 248 nm, τ_FWHM_ ≤ 25 ns), was used. The experiments were performed in water vapor atmosphere with ~50 Pa residual pressure. The laser beam was incident at 45 degrees on the target’s surface. The target-to-substrate separation distance was 5 cm. The laser pulse energy was set at 360 mJ (which corresponded to a fluence of 3.5 J/cm^2^), and was monitored by a Coherent system.

The ablated materials were transferred and collected onto medical grade Ti substrates and silicon wafers with dimensions of 10 × 10 × 0.2 and 10 × 10 × 0.4 mm^3^, respectively. Prior to introduction into the deposition chamber, to remove micro-impurities, the substrates were cleaned using a three-step protocol (in the order of acetone, ethyl alcohol, and deionized water, for ~20 min/step [42]) with an ultrasonic bath (model Elmasonic X-tra 30H). The cleaned substrates were dried by means of a jet of synthetic air (5.0 purity).

During the deposition process, the targets were continuously rotated (with a frequency of 0.75 Hz) and translated along two orthogonal axes to avoid piercing or the formation of craters, which have a negative influence on the quality of the synthesized layer as they deflect the laser plasma. To ensure the films’ uniformity, the exposure of a “fresh” surface to the action of each laser pulse was intended.

Using a PID-EXCEL controller, all substrates were maintained during the experiments at a constant temperature of 500 °C (heating and cooling rates of 20 and 10 °C/min, respectively).

Prior to the experiments, to eliminate the possibility of any contamination, a residual pressure of 10^−3^–10^−4^ Pa was attained in the reaction chamber. In addition, to clean the micro-impurities from the target surfaces, ~1500–2000 consecutive laser pulses were applied before each experiment. During this process, a screen-collector was positioned between the target and the substrate on the surface of which the ablated material condensed.

The number of laser pulses applied for the synthesis of each film was 5000, at a repetition rate of υ = 10 Hz.

After the deposition process, the fabricated thin films were submitted to thermal treatments (500 °C for 6 h), in ambient water vapors.

### 2.4. Physical–Chemical Characterization Methods of the Synthesized PLD Films

(**a**) The surface morphology of the MdHA thin films was investigated using a Gemini 500 field emission scanning electron microscope (Zeiss, Oberkochen, Germany). To determine the thickness of the synthesized thin films, Si (100) wafers were used. The reason for using Si substrates was both their flat surface and lack of any porosity. Moreover, they facilitate the preparation of samples for the cross-sectional microscopy as they can be easily cut according to a particular crystallographic direction.

(**b**) The chemical composition of the synthesized MdHA thin films was investigated using energy-dispersive X-ray spectroscopy (EDS). For these measurements, a Quatax EDS detector (Bruker, Billerica, MA, USA) attached to the Gemini 500 equipment was used. The elemental analyses were carried out in triplicate, in different, arbitrarily-selected areas of ~200 × 140 µm^2^ on the surface of the deposited layers.

(**c**) The crystalline structures of the PLD targets and derived films were analyzed by X-ray diffraction (XRD), with CuK_α_ radiation. The targets were investigated in Bragg–Brentano geometry using a Bruker D8 Advance diffractometer (Bruker AXS Advanced X-Ray Solutions GmbH, Karlsruhe, Germany) equipped with a high-efficiency LynxEye^TM^ 1D detector. The thin films were analyzed in grazing incidence geometry (GIXRD) using a Rigaku SmartLab 3 kW equipment (Rigaku Corporation, Tokyo, Japan) set up in a parallel beam mode at an incidence angle (α) of 2°. The XRD patterns of both the targets and the films were collected in the range of 2θ = 10–60°.

(**d**) To highlight the presence of functional groups and their structural arrangement in the synthesized thin films, Fourier transform infra-red (FTIR) spectroscopy investigations were carried out with a PerkinElmer BX Spectrum-Pike system in attenuated total reflectance (ATR) mode, using a Pike-MIRacle ZnSe/diamond crystal attachment with a diameter of 0.18 cm. The measurements were performed on Ti substrates at RT, with a resolution of 4 cm^−1^ and 64 scans/experiment.

(**e**) Microscopic characteristics, in terms of the surface roughness and surface energy of materials, have a key role in determining wetting behavior. By contact angle (CA) measurements, the hydrophobic/hydrophilic characteristics of the synthesized films were investigated. Static CAs were measured with a drop shape analysis system, model DSA100 (Krüss GmbH, Hamburg, Germany). The samples were placed onto a flat platform under the tip of a blunt-ended, stainless steel needle (outer diameter of 0.5 mm). The needle was attached to a syringe pump controlled by the DSA3^®^ PC software and used to drip precise volumes of liquid onto the test surface, for CA assessment. The volume and diameter of a single liquid droplet were ~1–3 μL and ~1–2 mm, respectively. For each individual sample, the analysis was performed on two different areas of the film’s surface. For the CA measurements, the experimental profile of the droplet was fitted with a second-degree polynomial, or with the equation of the circle. Next, the slope of the tangent to the droplet at the point of intersection with the line separating the liquid–solid–vapor interface was inferred. The angle between the video camera and the sample plane was ~2°. All wettability tests were performed at RT, in duplicate. Based on the measured CA values, the value of the surface free energy (SFE) was assessed. Two standard liquids (i.e., water and diiodomethane) were used, for which the surface tension (γ_water_ = 72.8 mN/m; γ_diiodomethane_ = 50.8 mN/m), the dispersion component, and the polar (γ_water_^d^ = 21.8 mN/m, γ_water_^p^ = 51 mN/m, γ_diiodomethane_ = 48.5 mN/m, and γ_diiodomethane_^p^ = 2.3 mN/m) values were known. Using the inferred CA values, both the polar and dispersion parts of the synthesized films, as well as the total SFE, were estimated by applying the Owens–Wendt method [49].

(**f**) The bonding strength (tensile) at the interface between the biofunctional film and the substrate is considered to be of key importance for the fabrication of high-quality implants and the long-term functioning and stability of these medical devices [6] in situ. Consequently, it is included as quality factor in the ISO 13779-2/2018 [50], which regulates the fabrication of load-bearing implant coatings. The adherence of the synthesized MdHA thin films on Ti substrates was estimated by the pull-out method. For these tests, a DFD Instruments^®^ PAThandy MICRO AT101 (maximum pull force of 1 kN) adherence instrument, equipped with stainless steel test elements (dollies) with diameters of 0.28 cm, was used. The dollies were glued to the thin films’ surface with a special E1100S single-part epoxy glue with a certified breaking strength of ~85 MPa. The stub surface was polished first, then ultrasonically cleaned with acetone and ethanol and dried in a nitrogen flow. After gluing, the MdHA thin films were dried in an oven (130 °C for 1 h). Prior to the investigations, four quality tests to control the bonding adhesive strength were performed using uncoated Ti substrates. The value of the adhesive strength, measured at the stainless-steel test element–Ti control interface, was 63 ± 0.5 MPa. Each dolly was pulled out vertically using a calibrated hydraulic pump. The extraction force was gradually increased until detachment occurred. For each thin film, the measurements were performed in quadruplicate and were in accordance with the ASTM D4541 and ISO 4624 standards. The mechanical adherence strength was determined from the measured failure force value divided by the area of the detached film surface. Mean values and standard deviation (SD) were computed.

### 2.5. Preliminary In Vitro Testing of the PLD FILMS

(**a**) The degradation of the MdHA films, derived from biogenic resources, was tested in accordance with the recommendations of the ISO 10993–14/2009: “Biological evaluation of medical devices—Part 14: Identification and quantification of degradation products from ceramics”. However, instead of the suggested Tris-HCl testing solution, the same medium as the one used for the further cytocompatibility tests was preferred—i.e., Dulbecco’s modified eagle medium (DMEM, Thermo Fisher Scientific, Waltham, MA, USA) supplemented with 10% fetal bovine serum (FBS, Thermo Fisher Scientific, Waltham, MA, USA)—since it enables a more homogeneous assessment across various in vitro evaluation protocols. Furthermore, the degradation testing was performed under homeostatic temperature (37 °C), pH (7.4), and (humidified) atmosphere conditions (5% of CO_2_). This is known to mitigate the medium evaporation and the necessity of its timely replacement/refreshing [51]. Sterile samples (obtained by dry-heating at 180 °C/1 h) were immersed in sealed vials with 0.5 mL of DMEM-FBS solution for 3, 7, and 28 days. After each time period, the samples were extracted, gently washed with distilled water, and then dried in an oven for 24 h at 37 °C. Each tested MdHA sample was weighted before and after the degradation testing with an analytical microbalance (Radwag, model MYA0.8/3.3Y, 1 µg readability). The mass variation in the MdHA thin films was thus evaluated and reported correspondingly as a mass loss or mass gain. Furthermore, the morphology changes of the thin film surfaces were inspected by scanning electron microscopy (SEM). All tests were performed in triplicate for each time interval.

(**b**) The apatite-forming ability of the MdHA thin films was evaluated in Kokubo’s simulated body fluid (SBF) solution by complying with the instructions of the ISO 23317/2014: “Implants for surgery: In vitro evaluation for apatite-forming ability of implant materials” [52,53,54]. The SBF solution was prepared in our laboratory by dissolving the chemical reagents (Sigma Aldrich, St. Louis, MI, USA) in 1000 mL of deionized water, and we obtained the following ion concentrations (10^−3^ mol): Na^+^: 142.0; K^+^: 5.0; Mg^2+^: 1.5; Ca^2+^: 2.5; Cl^−^: 147.8; HCO_3_^−^: 4.2; HPO_4_^2−^: 1.0; and SO_4_^2−^: 0.5. The sterile samples were placed in sealed vials with the SBF solution and introduced into a humidified incubator at 37 °C. The chosen testing immersion period was 30 days. Subsequently, the samples were removed from the solutions, gently washed with distilled water, and left to dry in a desiccator. After drying, the SBF-tested samples were investigated by SEM, EDS, and FTIR spectroscopy.

(**c**) The cytocompatibility of the simple and doped FB and SS thin films was tested in accordance with the ISO 10993–5/2009: “Biological evaluation of medical devices—Part 5: Tests for in vitro cytotoxicity”, on three cell lines. Thus, osteoblasts (G292) were used to test the osteogenic potential, whilst fibroblasts (NCTC L929) and epithelial (HeLa) cells were used to investigate their biocompatibility, according to the UNI EN ISO 10993 regulation for testing medical devices intended for human use [55]. In this respect, the use of epithelial cells was also important for the purpose of ensuring that the application range of these materials could be further extended to antimicrobial coatings for wound dressings and medical cloths. Osteoblasts were grown in McCoy′s 5a + 2 mM glutamine + 10% fetal bovine serum (FBS), according to the ATCC guidelines. Epithelial and fibroblast cells were grown in DMEM medium and supplemented with 10% fetal bovine serum (Sigma-Aldrich) and 1% Pen/Strep (penicillin/streptomycin solution, 50 µg/mL, Sigma-Aldrich) for 24 h at 37 °C and 95% humidity with 5% CO_2_. All cells were seeded at a density of 2 × 10^4^ cells/cm^2^ in 24-well plates onto the coated samples, previously sterilized under UV light, for 1 h. Cells were washed with saline solution (Sigma-Aldrich) and trypsin (trypsin-EDTA 0.25%, Thermo Fisher Scientific, Waltham, MA, USA), then counted using trypan blue and a hemocytometer. The MdHA thin films were co-cultured with the cells (seeding density of 5 × 10^5^ cells/well) for 24 h (37 °C, 95% humidity, 5% CO_2_).

(**c1**) MTT: one should note that the MTT compound [3-(4,5-dimethyltiazol-2-yl)-2,5-diphenyltetrazolium bromide] (Vybrant^®^ MTT Cell Proliferation Assay Kit, V-13154) is permeable to the membranes of living cells. After metabolizing the MTT compound, soluble formazan crystals were formed. This resulted in a solution (purple color) with an optical density that could be read at 550 nm. The MTT test allowed us to evaluate the viability and proliferation of living cells in the culture in the presence of the synthesized MdHA thin films. Cells were incubated with MTT reagent for 4 h at 37 °C and 95% humidity with 5% CO_2_. After incubation, the formazan crystals were solubilized with sodium dodecyl phosphate–chlorohydric acid for 18 h at 37 °C and 95% humidity with 5% CO_2_. The absorbance was measured using Mulsiskan FC (Thermo Fisher Scientific) at λ = 570 nm.

(**c2**) LDH: the cytotoxicity of the materials was evaluated using the lactate dehydrogenase (LDH) cytotoxicity detection kit (Roche, Basel, Switzerland). LDH is an enzyme which is present in the cytoplasm of all living animal cells. In the case of cell death, LDH was released into the culture medium; thus, the number of dead cells was directly proportional to the LDH activity. The LDH activity was measured in the supernatant using a Mulsiskan FC instrument (Thermo Fisher Scientific) at λ = 490 nm, with a wavelength reference of λ = 600 nm. After 24 h of incubation, the cell morphology was evaluated using an Olympus IX73 inverted fluorescence microscope (λex/em 488 nm/515 nm and λex/em 570 nm/602 nm).

(**c3**) ALP: the effect of biomaterials on the secretion of alkaline phosphatase (ALP) was investigated on the G292 cell line. The detection and quantification of ALP were performed using a colorimetric enzymatic assay (Thermo Fisher Scientific, Cat. No. ALP TR11320). The G292 osteoblasts were grown on the surface of Ti, as well as simple and doped FB and SS coatings, for 48 h. The cell supernatant was removed, and the samples covered with cells were placed into a new 24-well plate for measurements of the ALP activity. Cells were incubated with 4-nitrophenylphosphate substrate buffer to initiate the conversion into 4-nitrophenoxide. The absorbance of the yellow-colored product p-nitrophenol produced in the reaction was proportional to the amount of ALP present in the samples, and it was read at 405 nm on a Mulsiskan FC spectrophotometer (Thermo Fisher Scientific). Epithelial cells co-cultured with simple and doped FB and SS thin films were fixed with methanol (20 min at 4 °C) and subjected to staining for DNA (DAPI, 1 μg/mL) and plasma membranes (Cell Mask Deep Red, 8 μg/mL).

(**c4**) Osteocalcin concentration was measured using the Human Osteocalcin ELISA kit (cat no. RAB1073), following the manufacturer’s instructions, after 48 h of incubation of the osteoblasts with the tested materials.

(**d**) The investigation of the antimicrobial activity of simple and doped MdHA thin films was performed on standard microbial strains, i.e., *Staphylococcus aureus* (*S. aureus*, ATCC 25923), *Enterococcus faecalis* (*E. faecalis*, ATCC 29212), *Escherichia coli* (*E. coli*, ATCC 25922), *Pseudomonas aeruginosa* (*P. aeruginosa*, ATCC 27853), and *Candida albicans* (*C. albicans*, ATCC 26790). The simple and doped MdHA thin films, previously sterilized by UV exposure (30 min on each side), were put in contact with microbial suspensions with densities of 10^6^ CFU/mL (colony-forming units) in a liquid medium that maintained bacterial viability, but did not allow for its development. After different time intervals, i.e., initially (T_0_) and after 2, 4, 24, and 48 h, microbial viability was determined by performing serial dilutions and seeding on agar medium (i.e., Mueller–Hinton for bacteria and Saboraud for fungi) to calculate the number of CFUs after incubation for 18 h at 37 °C. All tests were performed in triplicate.

### 2.6. Statistical Analysis

All experiments were carried out in triplicate, in order for the statistical significance to be investigated. Data are shown as mean ± SD and were represented using GraphPad Prism software, version 9.4.1. The differences in biocompatibility measurements were calculated using the “Unpaired *t*-test”. The results of the microbiology testing were analyzed using the one-way ANOVA test with post hoc Bonferroni correction. The statistical significance levels were * *p* ≤ 0.05, ** *p* ≤ 0.01, and *** *p* ≤ 0.001.

## 3. Results and Discussion

### 3.1. SEM

**Figure 1:**
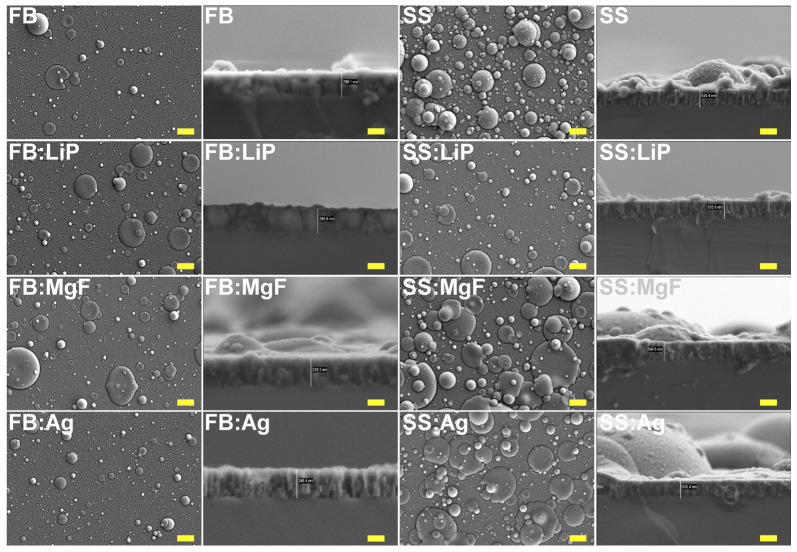
Top-view and cross-sectional SEM micrographs of simple and doped fish bone (FB) and seashell (SS)-derived hydroxyapatite thin films. Magnification bars: 2 µm (top-view) and 200 nm (cross-section).

Top-view SEM morphologies of simple and doped FB and SS thin films are comparatively presented in Figure 1, at a magnification of 5000×. One can observe that the films consisted of matrices with rough and irregular morphologies, made up of spheroidal particles, known as droplets, with different size distributions. The origin of these droplets and their relationship with the targets’ composition represent well-known characteristics of the PLD process [46]. Figure 1 shows that the density and diameters of the particles were much larger for the SS thin films in comparison to the FB ones. In the literature, it has been demonstrated that the random distribution and varied sizes of these particles determine a beneficial roughness for cell cultures by promoting the colonization of osteogenic cells in their microcavities, followed by their intense proliferation [56,57]. It is important to mention that no delamination phenomena or microcracks were observed in the synthesized films.

The cross-sectional SEM images (Figure 1) showed, in all cases, uniform coverage with columnar growth. The coatings’ thickness values (and, implicitly, the deposition rates) are presented in Table 2.

The deposition rate can also be influenced by the density and crystallinity of the target [58]. It has also been demonstrated that the addition of different ions influences both the properties of the coatings and the deposition rates [59]. Thus, the thickness variations observed in the case of the synthesized structures can be attributed mainly to the nature of the pristine material, but also to the different ablation rates of Li, Mg, and Ag. In general, when using the PLD technique, pure metals have higher ablation rates [60], which can justify a slightly higher deposition rate in the case of FB:Ag structures in comparison to simple FB ones.

### 3.2. EDS

**Figure 2:**
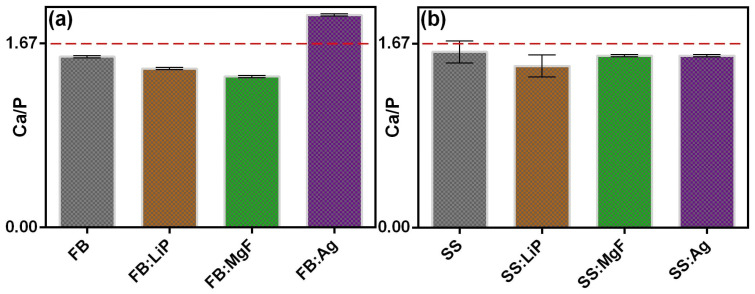
The value of the Ca/P ratio in the case of simple and doped FB (**a**) and SS (**b**) thin films.

In addition to the main constituents normally found in the chemical composition of HA (e.g., Ca, P, O), the existence of some trace elements present in the composition of the mineral phase of healthy bone, such as Mg, Si, and S, which play an important role in its functionality, was also highlighted. The presence of light elements (such as Li) could not be highlighted because of their low characteristic radiation energy, below the detection limit of this analysis technique. It is important to mention that no contaminating species were identified. The Ca/P ratio was also inferred, and the results are presented in Figure 2.

As one can observe (Figure 2), the deposited structures presented Ca/P values either higher (FB:Ag) or lower (the rest of the samples) than the theoretical value of stoichiometric HA (~1.67) (dashed line in Figure 2). It should be mentioned that these values were similar to those reported in the case of apatites of biological origin [37,56].

### 3.3. XRD

**Figure 3:**
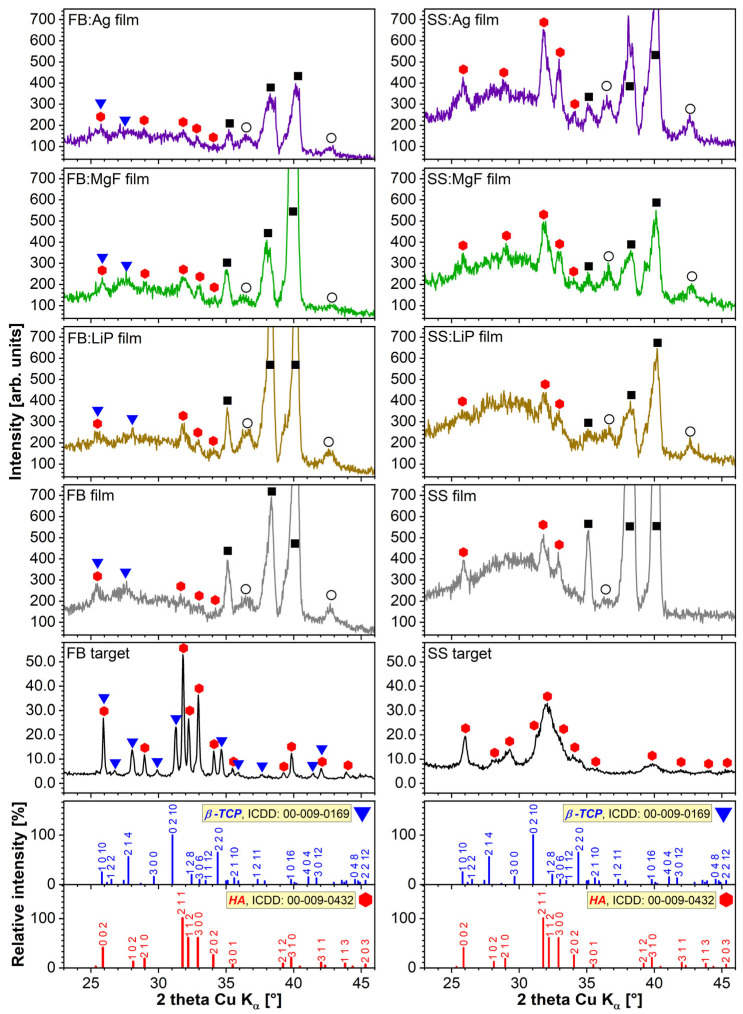
Comparison of the X-ray diffractograms recorded for the FB and SS source target materials (collected in Bragg–Brentano geometry) and of the simple and doped FB and SS thin films (collected in grazing incidence geometry). Symbols: 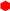
—hydroxyapatite; ▼—β-tricalcium phosphate; ■—titanium; ◯—TiO).

Figure 3 shows the diffractograms corresponding to the source FB and SS materials with respect to those of PLD-derived thin films. Marked differences were observed between the two CaP-based target materials: FB is well-crystallized and consists of a bi-phasic blend of HA (ICDD: 00-009-0432) and β-TCP (ICDD: 00-009-0169) phases, whilst SS is composed of single nanostructured (as indicated by the broad, less-defined diffraction maxima) HA phase. Biphasic calcium phosphate (BCP) materials are recognized for their superior osteoconduction potential with respect to pure, highly crystallized HA [57,61].

The GIXRD patterns of all PLD films were dominated by the intense maxima of the Ti substrate (indexed with reference file ICDD: 00-044-1294). The ubiquitous presence of a Ti oxidation product (i.e., TiO suboxide, ICDD: 01-086-2352) was noticed for all samples. The CaP-based PLD films yielded low-intensity broad peaks irrespective of the type of bioceramic matrix (i.e., FB or SS) or additive (i.e., Li_3_PO_4_, MgF_2_, or Ag), suggestive of their nano-structuring. Nevertheless, two important differences were noted between the FB and SS film series: (i) the FB films also contained, besides the dominant HA phase, traces of β-TCP; whilst (ii) the SS films, although they elicited higher-intensity HA peaks with respect to the FB films (due to the significantly higher thickness of the former), also presented an important amorphous (less-structured) component evidenced by the halo ranging between 2θ ≈ 23–33°. Thus, it can be argued that the main compositional features of the source materials were preserved.

### 3.4. FTIR Spectroscopy

**Figure 4:**
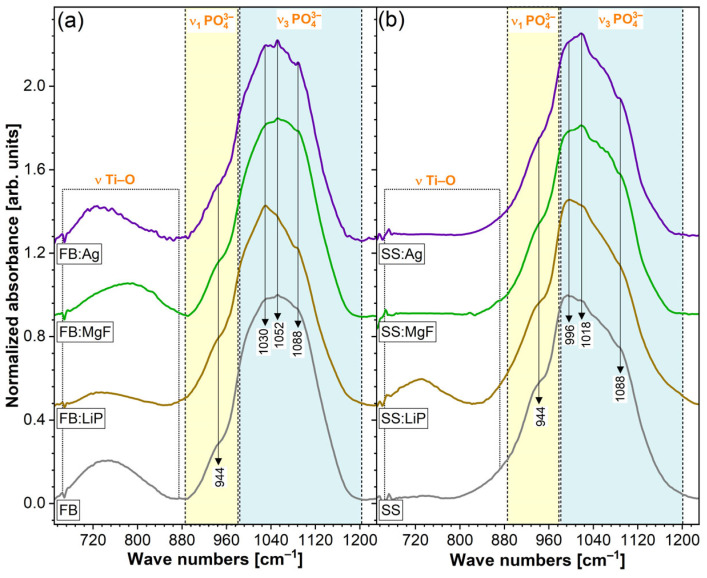
FTIR-ATR spectra of simple and doped FB (**a**) and SS (**b**) thin films.

Figure 4 represents the FTIR spectra of the simple and doped FB and SS thin films in a wave number range relevant for CaP (i.e., 1250–650 cm^−1^). The FB- and SS- samples elicited a major IR absorption maximum with definite shoulders characteristic of specific vibrational modes of orthophosphate functional units in CaPs, i.e., symmetric (*ν*_1_) stretching (990–930 cm^−1^) and triply degenerated asymmetric stretching (1200–990 cm^−1^) modes [4,62]. The FB-based samples presented more convoluted spectral envelopes with shallower shoulders, hinting towards a lower structural ordering and/or juxtaposition of (PO_4_)^3–^ bands stemming from more than one type of CaP phase (in good agreement with the XRD data). Moreover, a supplemental, lower-intensity IR band was evidenced in the wave number range of 850–650 cm^−1^, ascribed to vibrations of Ti–O bonds [43,63], furthermore confirming the XRD phase indexing.

### 3.5. CA and SFE

**Figure 5:**
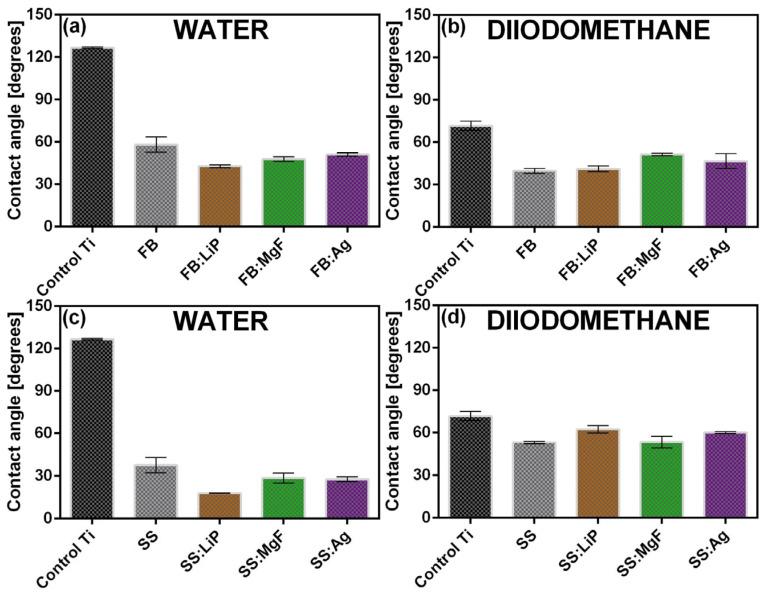
The contact angle values inferred in the case of Ti and simple and doped FB (**a**,**b**) and SS (**c**,**d**) thin films. Test liquids were water (**a**,**c**), and diiodomethane (**b**,**d**).

For comparison, the results of the CA measurements are presented in Figure 5. The surfaces of the Ti substrates demonstrated hydrophobic behavior (CA > 90°). Remarkably, the synthesis of the simple and doped FB and SS thin films radically changed the behavior of the surface towards hydrophilicity, with the lowest water CA values being recorded in the case of SS structures, i.e., ~(15–18°).

It was reported that a hydrophilic surface stimulated the adhesion of cells, along with their migration and proliferation, thus promoting a much faster bone regeneration process [57,64]. It is important to mention that the differences between the CA values recorded in the cases of two consecutive measurements, performed in different areas of the surfaces, were not significant. This result suggests, in agreement with the SEM results, a good uniformity of the synthesized thin films.

**Figure 6:**
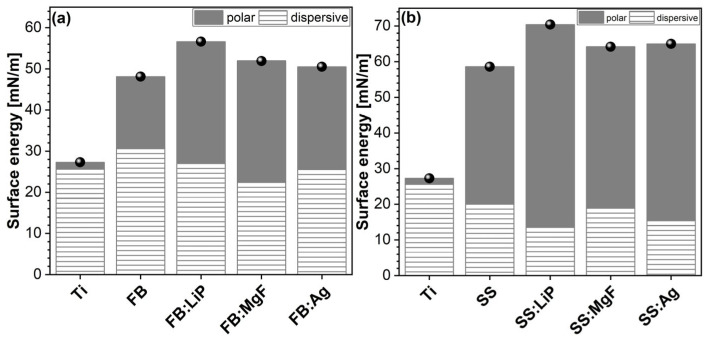
The values of the surface free energy calculated for Ti and simple and doped FB (**a**) and SS (**b**) thin films (γ^d^—hatched region, γ^p^—grey region).

The SFE values determined for the simple and doped FB and SS thin films are presented comparatively in Figure 6.

**Figure 7:**
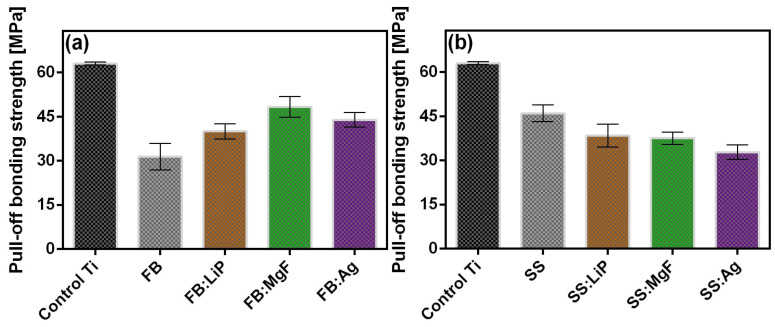
The adherence values at the film–Ti substrate interface for simple and doped FB (**a**) and SS (**b**) thin films.

Based on the obtained results, it can be stated that the calculated SFE values showed minor fluctuations, in the ranges of (45–57) mN/m (FB case) and (59–71) mN/m (SS case), which represents, structurally and morphologically, another piece of evidence for the good uniformity of deposited thin films. The SFE results showed that, in the case of the Ti control, the dispersive component (γ^d^) was the most pronounced (Figure 7, hatched region), while for the SS thin films, the polar component (γ^p^) increased significantly (Figure 7, gray region). This is an indication of an important change in the surface chemistry and electron density, and is most likely due to the presence of abundant polar groups on the structures’ surfaces (especially those doped with Li). This behavior, combined with the polar nature of the water droplet itself, determines an increased level of wettability.

### 3.6. Substrates Adherence

From a macroscopic point of view, all synthesized MdHA layers (simple and doped FB and SS thin films), were very adherent to the Ti substrates, as demonstrated by preliminary adhesion tests such as the finger test and application/removal of an adhesive tape (i.e., scotch test).

The measured values of adherence of the simple and doped FB and SS thin films are presented comparatively in Figure 7; these were calculated as mean ± standard deviation (SD). It should be mentioned that an event was considered only if the fracturing was of an adhesive type, i.e., when it occurred at the interface between the thin film and the Ti substrate.

The adherence values recorded for simple and doped FB and SS thin films were similar to those reported in the literature for biological-origin films synthesized by PLD [37,56]. It is also important to note that only adherence values higher than that imposed by the ISO 13779-2/2018 (i.e., >15 MPa) are considered acceptable for coatings intended for applications with high biomechanical loads [50]. Taking this criterion into account, the adherence values measured for simple and doped FB and SS thin films should be considered remarkable.

### 3.7. Preliminary In Vitro Tests

#### 3.7.1. Degradation Tests in Cell Culture Media

Table 3 shows both the mass losses (italic) and mass gains (bold) recorded for simple and doped FB and SS thin films after 3, 7, and 28 days of immersion in DMEM-FBS.

By evaluating the values presented in Table 3, a mass loss can be observed in the cases of all simple and doped FB thin films at 3 days of immersion in DMEM-FBS. Moreover, the solubilization process proved to be more intense in the case of FB thin films (a loss of 27.2%), followed by FB:MgF, FB:Ag, and FB:LiP. At 7 days, the solubilization process continued, but it seemed to be accompanied by a precipitation of CaP, a behavior emerging from the evolution of mass values (lower mass losses being recorded with respect to those obtained after 3 days of immersion). This time, the mass losses were higher in the case of FB:LiP thin films (15.1%). After 28 days, for both simple and doped FB thin films, noteworthy mass increases were recorded, the most important one (31.5%) being observed for the FB thin films (Table 3). The calculated values suggest, once again, the occurrence of the precipitation of CaP phases, which has previously been reported when testing in homeostatic conditions in such complex inorganic–organic synthetic physiological fluids [54,65].

Regarding the simple and doped SS thin films, only mass increases were evident for all three time intervals, except for the SS:MgF films, for which a decrease of 0.9% was recorded after 3 days. Thus, after 3 and 7 days of immersion in DMEM-FBS, the most important mass increases were recorded for SS:LiP (10.1% vs. 14.8%). However, after 28 days, the highest value of the mass increase was evident in the case of SS thin films (41.7%)—the highest among all investigated MdHA layers. These results suggest that the SS thin films induced more pronounced CaP in-growths. This can be linked to the higher content of the HA phase in the SS-based samples, known to foster increased apatite-forming ability under biomimetic conditions [66].

**Figure 8:**
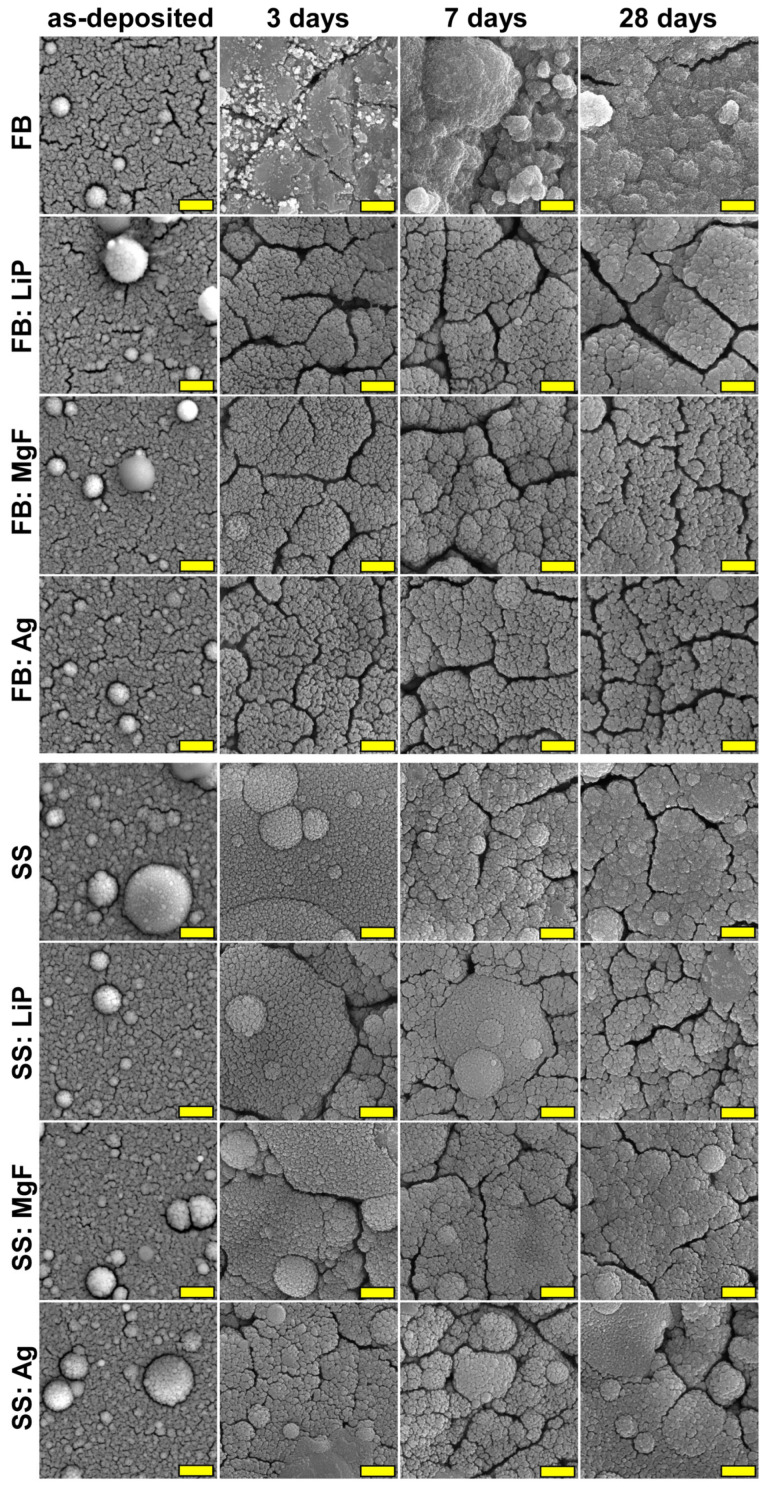
SEM images of the surface of the simple and doped FB- and SS-based thin films before and after testing in the complete DMEM-FBS solution for different time intervals (3, 7, and 28 days). Magnification bar: 500 nm.

The SEM investigations (Figure 8) indicated that morphological changes occur upon immersion in DMEM-FBS, the most marked being recorded in the case of simple FB and SS thin films, in good accordance with the mass loss/gain results (Table 3). These sample topographies were radically modified, with the films’ surfaces consisting of blankets of very fine crystals (smaller (for SS) or much smaller (for FB) than in the case of the as-deposited films), which seemed to aggregate into randomly-distributed spherulites. This type of morphology is characteristic of biomimetically-grown CaP deposits [52,54], and it prompted the apatite-forming ability test, which was performed in agreement with the ISO 23317/2014 (the results of which are presented hereafter).

#### 3.7.2. Apatite-Forming Ability Assays

**Figure 9:**
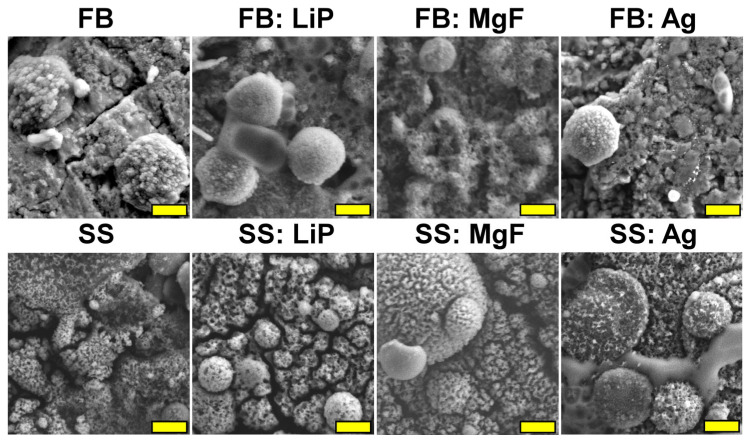
SEM images of the simple and doped FB- and SS-based thin films immersed in SBF for 30 days. Magnification bar: 500 nm.

Figure 9 shows typical SEM images of simple and doped FB and SS thin films after 30 days of SBF immersion. With respect to the as-deposited films, all SBF-tested samples exhibited radically altered morphology, with the surface of the samples being covered by acicular nano-crystals which formed spherulite aggregates (seemingly more pronounced than those noticed in the case of DMEM-FBS testing at the 28-day timepoint). As previously highlighted, this is typical for biomimetic CaP-layers developed in physiological environments [52,54,57,67]).

One should note that the microcracks observed on the surfaces of thin films tested in both DMEM-FBS (Figure 8) and SBF (Figure 9) were induced by the drying process after the sample’s extraction from the testing medium.

EDS investigations (Figure S1) demonstrated that the Ca/P ratios in the case of chemically-grown apatite layers on the surface of the synthesized thin films presented values in the ~1.3–2.2 range, similar to values of biological origin HA [37,56]. It should be emphasized here that non-stoichiometric HA, which presents high solubility rates, is similar to biological apatite and, thus, is more suitable for use in implant coating applications [68]. In addition, these layers of porous HA have the role of improving both the osseointegration and the osteoinduction properties [69].

**Figure 10:**
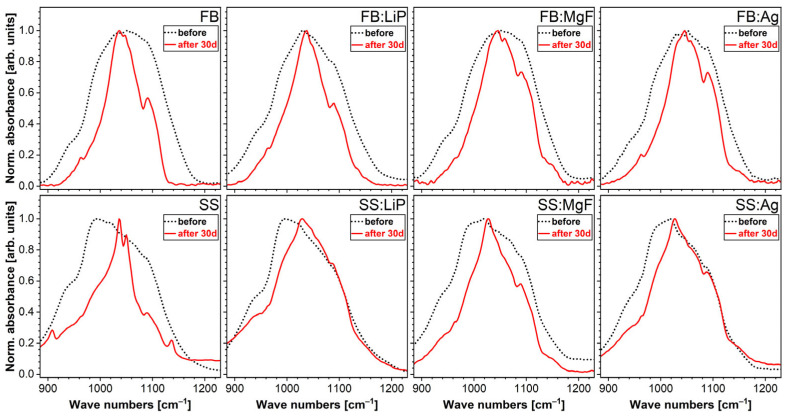
FTIR-ATR spectra of simple and doped FB and SS thin films immersed in SBF for 30 days.

Further evidence in support of the SEM- and EDS-derived hypothesis was provided by the FTIR-ATR spectroscopy (Figure 10). The spectra were recorded in the higher-intensity region of the symmetric and asymmetric stretching vibrational modes of phosphate units, in order to be able to discern structural changes (if any) with more ease. Furthermore, in the ATR mode, the FTIR spectroscopy analyses were more sensible to the sample surfaces and their superficial modifications (with respect to the transmission mode). In the case of all SBF-immersed samples, an evident narrowing of the absorption bands was noticed, indicating the formation of a CaP phase with improved structural ordering on the surfaces of the samples. The most radical modification was recorded for the simple SS sample, which agreed well with the weighting data, showing, in this case, the greatest mass gain.

### 3.8. Cytocompatibility Assays

Considering the targeted applications of these materials for dental implants, a cytocompatibility evaluation was performed using quantitative and qualitative assays on three types of cells, i.e., osteoblasts, fibroblasts, and epithelial cells, an implant material will interact with [70]. Thus, in contrast with orthopedic bone implants, oral ones must integrate not only into the surrounding bone, but also into the adjacent soft tissues [71].

**Figure 11:**
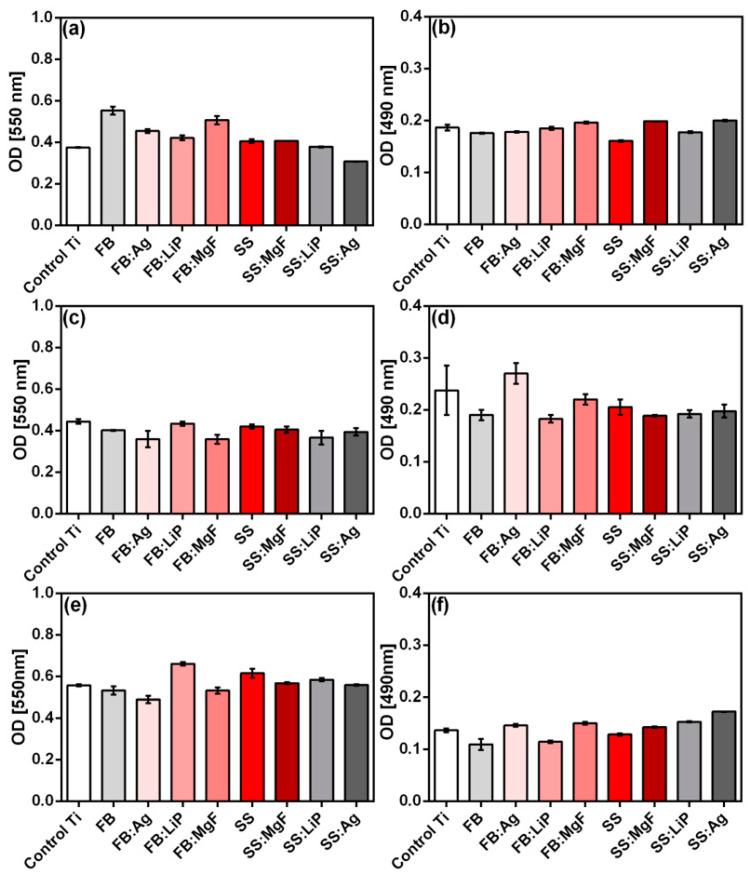
MTT (**a**,**c**,**e**) and LDH (**b**,**d**,**f**) biocompatibility tests (24 h) in the case of simple and doped FB and SS thin films, using osteoblast (**a**,**b**), fibroblast (**c**,**d**), and HeLa (**e**,**f**) cells.

Concerning the osteoblast cells, among all the tested structures, the FB thin films showed the best cytocompatibility, which was characterized by increased values of the MTT test correlating with low LDH level values (Figure 11a,b). Of all the tested samples, only SS:Ag induced a slight decrease in the cellular viability revealed by the MTT test (Figure 11a), which was also correlated with sub-confluent cellular monolayers evidenced by the phase contrast microscopic examination (Figure S2). Besides SS:Ag, sub-confluent monolayers were obtained on the SS:MgF sample. These two materials were also associated with the most elevated levels of LDH (Figure 11b), suggesting the induction of cellular damage.

As the LDH test results illustrate (Figure 11b), none of the simple or doped thin films derived from FB or SS were cytotoxic.

Regarding the NCTC fibroblasts (Figure 11c,d), the behavior of these cells on the surface of the obtained samples was different compared to that of osteoblasts. All samples induced a slight, albeit not statistically significant, decrease in the cellular viability assessed by MTT (Figure 11c). However, in the LDH assay (Figure 11d), all tested samples, except FB:Ag, showed increased cytocompatibility as compared to the Ti control. These results were also sustained by the fluorescence microscopy images using the Live/Dead staining, showing exclusively the presence of live (green) cells grown on the surface of the obtained samples, with morphological features similar to those of the control Ti (Figure S3). Taken together, the results of the MTT and LDH assays suggest that the FB:LiP thin films are the most cytocompatible in relation with fibroblast cells, characterized by increased values of the MTT test and correlated with low values of LDH (Figure 11c,d).

Concerning the epithelial HeLa cells (Figure 11e,f), the samples containing Ag and MgF (i.e., FB:Ag and FB:MgF) were slightly cytotoxic, as revealed by the lower MTT (Figure 11e), which was correlated with higher LDH values (Figure 11f). Taken together, the results of the MTT and LDH tests indicate that the FB:LiP material exhibited the best cytocompatibility (Figure 11e,f). The cytocompatibility evaluation of HeLa epithelial cells was also confirmed by fluorescence microscopy using DAPI and Cell Mask DeepRed, showing that the cells developed on the tested samples exhibited morphological features similar to those developed on the control surface (i.e., polystyrene surface of the culture dish) (Figure S4).

### 3.9. Influence of the Obtained Samples on the Production of Osteogenic Markers

#### 3.9.1. Alkaline Phosphatase

**Figure 12:**
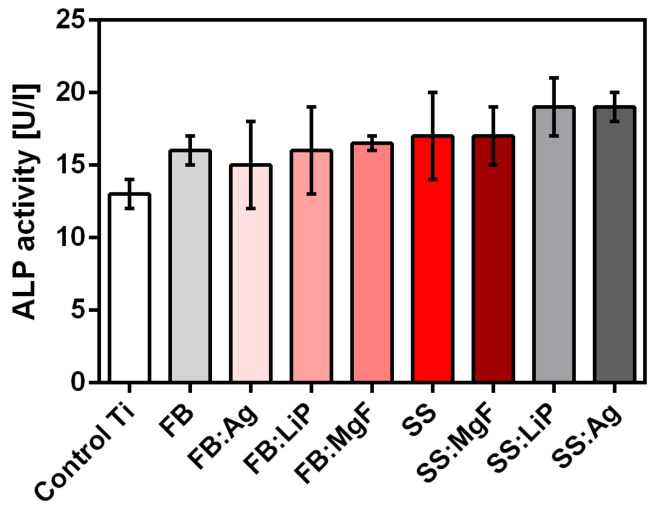
The effect of simple and doped FB and SS thin films on the alkaline phosphatase (ALP), 48 h after incubation.

ALP secreted by osteoblasts represents an early osteogenic marker for bone formation and bone calcification, inducing an elevated phosphate level at the cellular surface during bone mineralization [72]. Previous studies have shown that nanostructured surfaces can promote ALP production, improving the biocompatibility of the implant surface [73]. Therefore, we assessed the production of ALP by osteoblast cells grown on the obtained samples. Compared to uncoated Ti, all simple and doped FB and SS thin films stimulated the secretion of ALP after 48 h of incubation, with the highest levels being recorded for SS:Ag and SS:LiP (Figure 12).

#### 3.9.2. Osteocalcin

**Figure 13:**
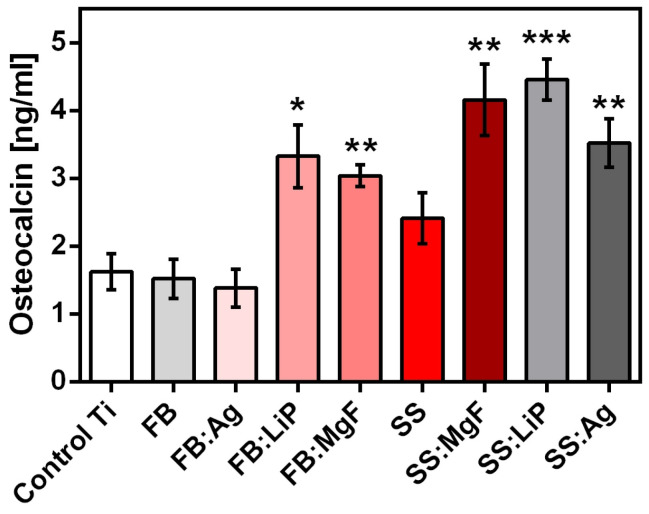
The effect of simple and doped FB and SS thin films on the osteocalcin concentration, 48 h after incubation (* *p* < 0.05; ** *p* < 0.01; *** *p* < 0.0001).

Osteocalcin (bone γ-carboxy glutamic acid), the most important non-collagen bone protein, which is also found in teeth, is an osteogenesis marker mainly produced by osteoblasts [74]. In order to quantify osteocalcin (a regulator of bone mineralization) levels after incubation with the materials, an additional experiment was performed and the obtained results are shown in Figure 13.

One should emphasize that the results obtained after osteocalcin quantification (Figure 13) were in good accordance with the ALP levels (Figure 12).

### 3.10. Antimicrobial Activity

Peri-implantitis represents one of the most frequent complications leading to the loss of an implant. It requires the development of novel materials with improved resistance to microbial colonization and biofilm development [75]. Peri-implantitis is a polymicrobial infection produced by microbial species which are more diverse and different from those involved in periodontitis [76]. Among them, *S. aureus* plays an important role in the development of peri-implantitis [76], probably due to its high adhesion capacity to Ti surface [77]. Enteric bacteria, *Pseudomonas* sp., *Enterococcus* sp., and *Candida* sp. were also frequently found in peri-implantitis lesions, suggesting that these microorganisms could be involved in the infection of soft and hard tissues surrounding the implant [78,79,80,81]. Although anaerobic species are also key players in dental implant infections, due to technical difficulties in working with anaerobic species, the antimicrobial activity of the simple and doped FB and SS thin films was investigated in this study on facultatively anaerobic Gram-positive and Gram-negative bacteria and fungal strains known to be involved in the etiology of periprosthetic polymicrobial infections (Table 4, Figures S5 and S6).

In the case of the *E. coli* strain, the antibacterial effect of the tested materials became evident from T_0_, as indicated by the logCFU/mL values recovered from different samples, in comparison with the bare Ti used as the control (Table 4, Figure S5). Statistically significant inhibition was, however, noticed only for FB:LiP, FB:MgF, and SS:Ag. After 4 h, the majority of the tested materials, with the exception of FB:Ag, SS, and SS:LiP, and after 24 h, all of the tested materials, had significantly decreased the logCFU/mL, while at 48 h, the antibacterial protection had been reduced, with a significant decrease in viable cells noticed for FB:MgF, FB:Ag, FB:LiP, and SS:LiP (Table 4, Figure S5).

In the case of the *P. aeruginosa* strain, at T_0_, similar colonization values were obtained for all tested materials (Table 4, Figure S6). After 2 h, although a slight decrease in the logCFU/mL was noticed, the results were not statistically significant. After 4 h of incubation, all tested materials significantly decreased the levels of *P. aeruginosa* as compared to the control Ti (Table 4, Figure S6). The FB and FB:Ag thin films significantly inhibited the growth of *P. aeruginosa* after 24 h of incubation (a 3–4 log reduction as compared to the control Ti: Table 4, Figure S6). After 48 h of incubation, all of the tested materials indicated a lower degree of colonization with *P. aeruginosa*, but statistically significant values were recorded only in the case of FB:Ag, FB:LiP, FB:MgF, SS:MgF, and SS:Ag thin films (Table 4, Figure S6).

In the case of Gram-positive strains, less competitive behavior was recorded in the tested materials in comparison with the Gram-negative ones. For the *S. aureus* strain (Table 5, Figure S7), at T_0_ and at 2 and 4 h, similar levels of colonization were obtained for all tested materials.

Among all the tested materials, FB:LiP, SS:Ag, SS:LiP, and SS:MgF thin films demonstrated statistically significant levels of growth inhibition (as compared to the control Ti) of the *S. aureus* strain after 24 h of incubation (Table 5, Figure S7).

In the case of the *E. faecalis* strain, besides FB:LiP (T_0_), FB:MgF (2 h), and FB (24 h), none of the tested materials significantly inhibited the bacterial growth in comparison with the control Ti (Figure S8).

In the case of the fungal strain (Table 5, Figure S9), surprisingly, all of the tested materials had significantly inhibited the biofilm after 48 h in comparison with bare Ti, although no significant inhibition was recorded at the previous tested intervals (except for SS, at T_0_ and 2 h).

The obtained results indicated that the addition of LiP, MgF, or Ag to FB and SS thin films maintained—and, in many cases, even increased—their anti-biofilm characteristics, with the effect occurring in earlier steps of biofilm development and, in some cases, being extended to 48 h. Considering that the method used to investigate the microbial adhesion and biofilm evolution on the synthesized layers implicated the quantification of the viable cells that adhered to the films’ surface, the hypothesis that the synthesized thin films exhibited microbicidal activity, therefore killing the cells before or after their contact with the coated surfaces, could explain the decreased number of biofilm-embedded cells. One should note that this hypothesis is supported by results showing the superior microbicidal activity of simple and doped FB and SS thin films in comparison to the control and Ti, mainly against the *S. aureus*, *E. faecalis,* and *C. albicans* strains.

Even though there are some papers which have reported on biological HA-type coatings obtained by PVD techniques, it should be emphasized that the current preliminary results were the first to be reported on MdHA structures fabricated by PLD. One major advantage of these MdHA coatings is related to the fact that they are synthesized from low-cost, sustainable resources, which are available in huge amounts. Taking into consideration some important demonstrated characteristics, such as hydrophilic behavior, high bonding strength values, low toxicity, and persistent protection against bacterial colonization and fungal biofilm development, these MdHA coatings should be advanced as viable alternatives to synthetic HA for dental implant applications. Even though excellent preliminary, short-term results were obtained, in dentistry, many complex problems might arise; therefore, long-term investigations should also be considered to be of keen importance.

HA was also demonstrated to possess an efficiency strong enough to capture heavy metals by changing cations or adsorbing unchangeable ones onto the crystal surface [82,83]. As a consequence, the amount of possible toxic elements which might be present in the inorganic components of fish bones and/or seashells should also be considered. In this respect, in the case of implants used for various medical applications, the presence of heavy metals (i.e., Cu, Cd, Pb, and/or Hg) in fish bones and/or seashells must be below the concentration limit allowed for inorganic bones [84].

Last, but not least, proper management of the oral microbiota can be considered one important factor that can positively influence the success rate of a dental implant. As proactive and home-based approaches, there are several minimally invasive protocols that must be followed/implemented by every patient to control eubiosis: the removal of bacterial plaque by toothbrushes and interdental subsidies; reducing the bacterial load using probiotics and paraprobiotics (i.e., chewing gum, toothpastes, and mousses); and healing inflammation with the help of natural substances [85,86].

## 4. Conclusions

In this study, we reported on the synthesis by PLD of thin layers of hydroxyapatite (HA) of marine origin (MdHA), derived from salmon fish bones (FB) and seashells (SS), both simple and doped (with Li_3_PO_4_, MgF_2_, or Ag, at a concentration of 0.5 wt.%). The fabricated thin films were investigated from morphological, compositional, structural, wettability, and mechanical points of view. In addition, all synthesized thin films were analyzed in vitro by immersion in biological fluids, cell culture assays, and antimicrobial tests.

SEM investigations indicated the existence of surfaces with rough and irregular morphologies, made of particulates (with dimensions of the order of hundreds of nm), which were reported as friendly environments for cells’ attachment and growth. The results of the XRD and FTIR spectroscopy analyses evidenced different degrees of crystallinity for the synthesized structures, generally influenced by the dopant’s nature and concentration, as well as by the pristine material. For Ti, hydrophobic behavior was observed in comparison to the simple and doped FB and SS thin films, for which strong hydrophilic behavior was detected. Pull-out tests indicated very adherent coatings with values more than three times higher than those imposed by current ISO recommendation.

After only three days of immersion in SBF, the surfaces of the synthesized thin films were uniformly covered by acicular formations. In addition, FTIR spectroscopy investigations indicated a change of the spectral envelopes by narrowing of the absorption bands, which was correlated with the in-growth of biomimetic HA (bio-mineralization capacity). The results of DMEM-FBS immersion suggested that the simple and doped FB and SS thin films were dominated by the CaP precipitation phenomenon, which was more pronounced in the case of films with higher contents of the bioactive HA phase.

The best cytocompatibility was obtained in the case of FB (on osteoblast cells) and FB:LiP (on fibroblast and epithelial cells) thin films. None of the tested layers was cytotoxic, and compared with control Ti, all materials stimulated the secretion of ALP after 48 h of incubation.

All investigated materials showed antimicrobial activity, the most intense effect being obtained in the case of FB:Ag structures. This was most likely due to the presence of the Ag dopant, which, when used in low concentrations, was reported to be a strong barrier against adhesion and contamination with micro-organisms.

The fabrication of competitive metal implant coatings from renewable resources proved to be efficient not only in ensuring a high adherence to the implant’s surface and a good cytocompatibility, but also in representing protective barriers against microbial colonization along with proactive action for long-term maintenance. Thus, this process can constitute an important step in the successful development of a new generation of coatings for dental implants.

## Figures and Tables

**Table 1:** Sample codes of all the materials utilized and their descriptions.

Sample Code	Description	Sample Code	Description
Ti	Titanium (deposition substrate)	SS	HA obtained from seashells
FB	HA obtained from fish bones	SS:LiP	HA obtained from seashells and doped with 0.5 wt.% Li_3_PO_4_
FB:LiP	HA obtained from fish bones and doped with 0.5 wt.% Li_3_PO_4_		
FB:MgF	HA obtained from fish bones and doped with 0.5 wt.% MgF_2_	SS:MgF	HA obtained from seashells and doped with 0.5 wt.% MgF_2_
FB:Ag	HA obtained from fish bones and doped with 0.5 wt.% Ag	SS:Ag	HA obtained from seashells and doped with 0.5 wt.% Ag

**Table 2:** Mean thicknesses and deposition rates of simple and doped FB and SS thin films.

Sample Code	Mean Thickness ± SD [nm]	Deposition Rate [nm/pulse]	Sample Code	Mean Thickness ± SD [nm]	Deposition Rate [nm/pulse]
FB	285.9 ± 3.2	5.7×10^−2^	SS	517.7 ± 18.3	10.3 ×10^−2^
FB:LiP	288.1 ± 3.2	5.8 ×10^−2^	SS:LiP	540.6 ± 6.6	10.8 ×10^−2^
FB:MgF	328.3 ± 0.1	6.6 ×10^−2^	SS:MgF	562.1 ± 10.5	11.2 ×10^−2^
FB:Ag	337.7 ± 12.6	6.7 ×10^−2^	SS:Ag	529.5 ± 1.34	10.6 ×10^−2^

**Table 3:** The mass losses (in *italics*) and gains (in **bold**) obtained for simple and doped FB and SS thin films after immersion in DMEM-FBS for 3, 7, and 28 days. The values were calculated as percentages (%) from the initially deposited film masses (before immersion in DMEM-FBS).

	FB	FB:LiP	FB:MgF	FB:Ag	SS	SS:LiP	SS:MgF	SS:Ag
3 days	*27.2*	*19.2*	*24.5*	*23.5*	**3.2**	**10.1**	*0.9*	**6.2**
7 days	**10.3**	*15.1*	*14*	*10.1*	**5.4**	**14.8**	**0.6**	**9.9**
28 days	**31.5**	**11.2**	**16.5**	**26.5**	**41.7**	**35.7**	**3.2**	**11.6**

**Table 4:** Representation of the statistically significant inhibitory effect exhibited by different samples against the adherence and growth of *E. coli* and *P. aeruginosa* (in **bold**) at different incubation times (* *p* < 0.05; ** *p* < 0.01; *** *p* < 0.0001).

*E. coli*/*P. aeruginosa*	T_0_		2 h		4 h		24 h		48 h	
FB	–	–	–	–	**	***	***	**	–	–
FB:Ag	–	–	–	–	–	***	***	**	**	***
FB:LiP	*	–	–	–	**	***	***	–	**	**
FB:MgF	–	–	*	–	***	***	***	–	***	**
SS	–	–	–	–	–	***	***	–	–	–
SS:MgF	–	–	–	–	**	***	***	–	–	**
SS:LiP	–	–	–	–	–	***	***	–	**	–
SS:Ag	–	–	*	–	*	***	***	–	–	***

**Table 5:** Representation of the statistically significant inhibitory effect exhibited by different samples against the Gram-positive (i.e., *S. aureus)* and fungal (i.e., *C. albicans)* (in **bold**) strains’ adherence and growth at different incubation times (** *p* < 0.01; *** *p* < 0.0001).

*S. aureus*/*C. albicans*	T_0_		2 h		4 h		24 h		48 h	
FB	–	–	–	–	–	–	–	–	–	***
FB:Ag	–	–	–	–	–	–	–	–	–	***
FB:LiP	–	–	–	–	–	–	***	–	–	***
FB:MgF	–	–	–	–	–	–	–	–	–	***
SS	–	**	***	–	–	–	–	–	–	***
SS:MgF	–	–	–	–	–	–	***	–	–	***
SS:LiP	–	–	–	–	–	–	***	–	–	***
SS:Ag	–	–	***	–	–	–	***	–	–	***

## Data Availability

Not applicable.

## Supplementary Materials

The following supporting information can be downloaded at: https://www.mdpi.com/article/10.3390/pharmaceutics15041294/s1, Figure S1: The values of the Ca/P ratio corresponding to the simple and doped FB si SS structures after immersion in SBF for 30 days. Figure S2: Contrast phase microscopy images of control Ti and simple and doped FB and SS thin films’ biocompatibility, tested on osteoblast cells (G292). Magnification bar: 100 μm. Figure S3: Fluorescence microscopy images of “Live/Dead” test, performed with NCTC L929 fibroblast cells on control Ti and simple and doped FB and SS thin films. Magnification bar: 100 μm. Figure S4: Fluorescence microscopy images acquired for control Ti and simple and doped FB and SS thin films using epithelial cells (HeLa). Magnification bar: 100 μm. Figure S5: Number of microbial viable cells recovered from the biofilms developing on control Ti and simple and doped FB and SS thin films at T_0_ (a), 2 h (b), 4 h (c), 24 h (d), and 48 h (e) using the *E. coli* strain (* *p* < 0.05; ** *p* < 0.01; *** *p* < 0.0001). Figure S6. Number of microbial viable cells recovered from the biofilms developing on control Ti and simple and doped FB and SS thin films at T_0_ (a), 2 h (b), 4 h (c), 24 h (d), and 48 h (e) using the *P. aeruginosa* strain (** *p* < 0.01; *** *p* < 0.0001). Figure S7: Number of microbial viable cells recovered from the biofilms developing on control Ti and simple and doped FB and SS thin films at T_0_ (a), 2 h (b), 4 h (c), 24 h (d), and 48 h (e) using the *S. aureus* strain (*** *p* < 0.0001). Figure S8: Number of microbial viable cells recovered from the biofilms developing on control Ti and simple and doped FB and SS thin films at T_0_ (a), 2 h (b), 4 h (c), 24 h (d), and 48 h (e) using the *E. faecalis* strain (* *p* < 0.05; *** *p* < 0.0001). Figure S9: Number of microbial viable cells recovered from the biofilms developing on control Ti and simple and doped FB and SS thin films at T_0_ (a), 2 h (b), 4 h (c), 24 h (d), and 48 h (e) using the *C. albicans* strain (** *p* < 0.01; *** *p* < 0.0001).
