# Supplementary material for: Hydroxyapatite Thin Films of Marine Origin as Sustainable Candidates for Dental Implants
Source: Pharmaceutics. 2023 Apr 20;15(4):1294. doi: 10.3390/pharmaceutics15041294 (PMC10142946; doi:10.3390/pharmaceutics15041294)
Supplement: Supplementary file 1 [file pharmaceutics-15-01294-s001.zip › pharmaceutics-2340436-supplementary.pdf]

pharmaceutics

MDPI

Article

Hydroxyapatite Thin Films of Marine Origin as Sustainable Candidates for Dental Implants

Gabriela Dorcioman [1], Valentina Grumezescu [1], George E. Stan [2], Mariana Carmen Chifiriuc [3,4,5], Gratiela Pircalabioru Gradisteanu [4,6,*], Florin Miculescu [7], Elena Matei [2], Gianina Popescu-Pelin [1], Irina Zgura [2], Valentin Craciun [1], Faik Nüzhet Oktar [8,9] and Liviu Duta [1,*]

[1] Lasers Department, National Institute for Lasers, Plasma and Radiation Physics, 077125 Magurele, Romania
[2] National Institute of Materials Physics, 077125 Magurele, Romania
[3] Department of Microbiology, Faculty of Biology, University of Bucharest, 060101 Bucharest, Romania
[4] Earth, Environmental and Life Sciences Division, Research Institute of the University of Bucharest (ICUB), 060101 Bucharest, Romania
[5] Romanian Academy, 010071 Bucharest, Romania
[6] Academy of Romanian Scientists, 051157 Bucharest, Romania
[7] Faculty of Materials Science and Engineering, Politehnica University of Bucharest, 060042 Bucharest, Romania
[8] Department of Bioengineering, Faculty of Engineering, University of Marmara, 34722 Istanbul, Turkey
[9] Advanced Nanomaterials Research Laboratory (ANRL), University of Marmara, 34722 Istanbul, Turkey
* Correspondence: gratiela.gradisteanu@icub.unibuc.ro (G.P.G.); liviu.duta@inflpr.ro (L.D.); Tel.: +40-(0)7-2766-8664 (G.P.); +40-(0)2-1457-4450 (ext. 2023) (L.D.)

1. Preliminary *in vitro* testing

1.1 Apatite-forming ability

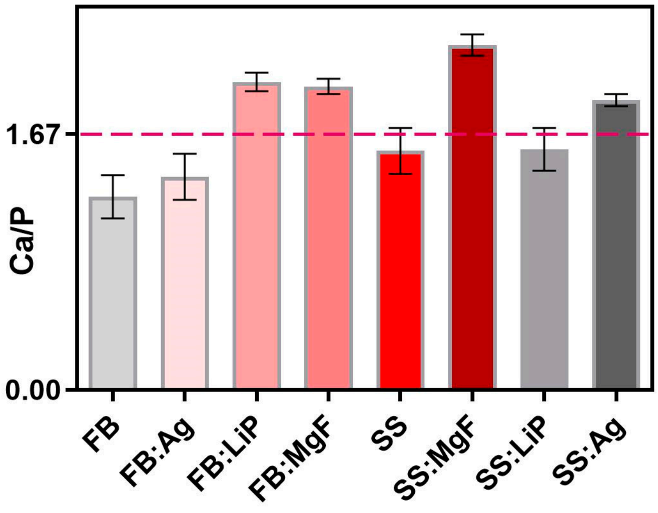

Figure S1. The values of the Ca/P ratio corresponding to the simple and doped FB si SS structures after immersion in SBF for 30 days. Note: dashed line represents the theoretical value of stoichiometric HA (~1.67).

2. Cytocompatibility Assays

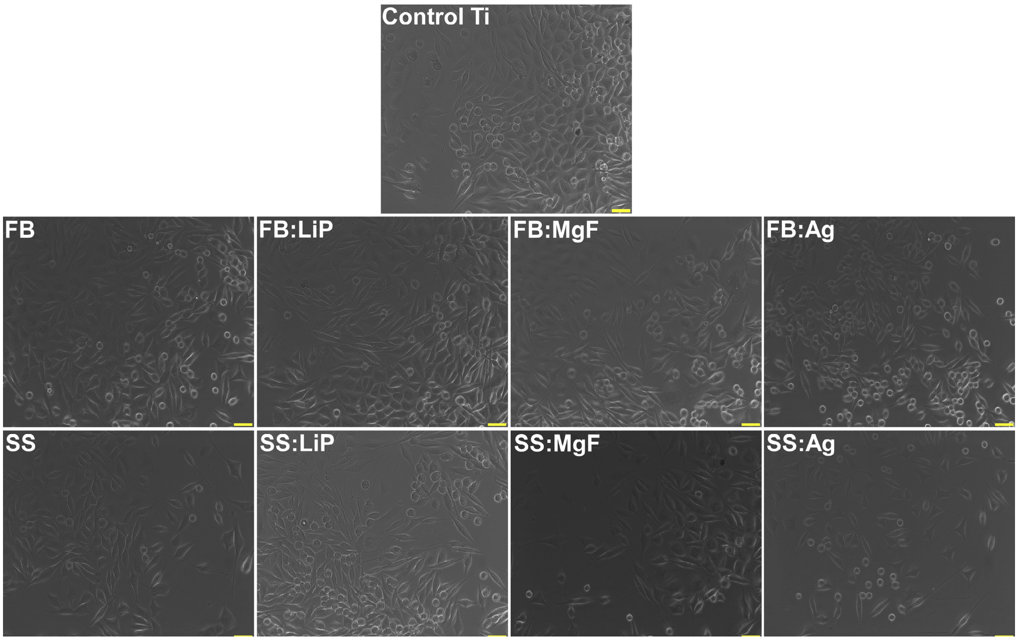

Figure S2. Contrast phase microscopy images of control Ti and simple and doped FB and SS thin films' biocompatibility, tested on osteoblast cells (G292). Magnification bar: 100 µm.

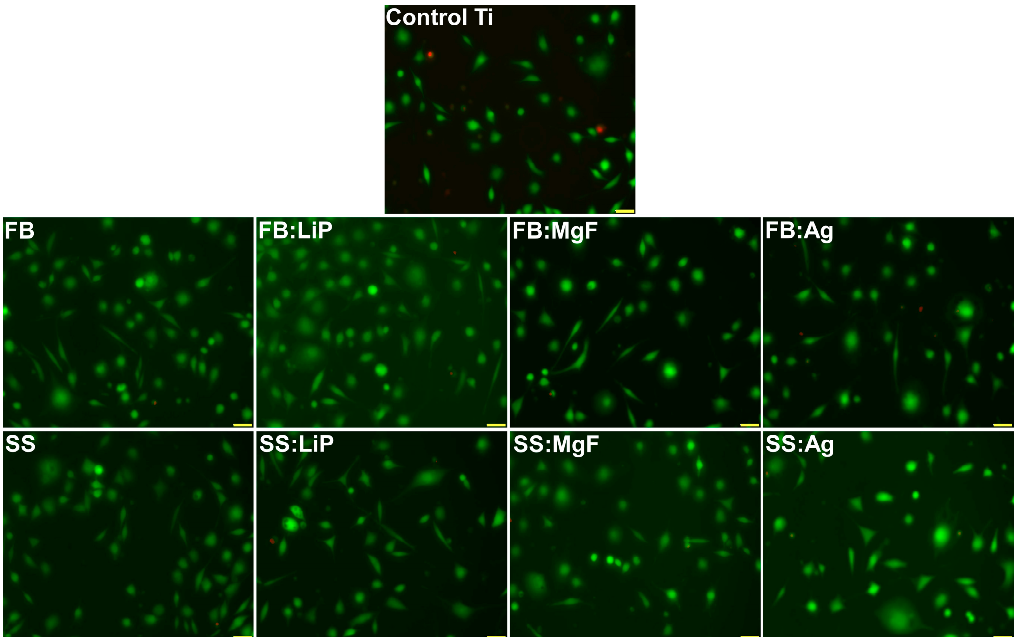

Figure S3. Fluorescence microscopy images of "Live/Dead" test, performed with NCTC L929 fibroblast cells on control Ti and simple and doped FB and SS thin films. Magnification bar: 100 µm.

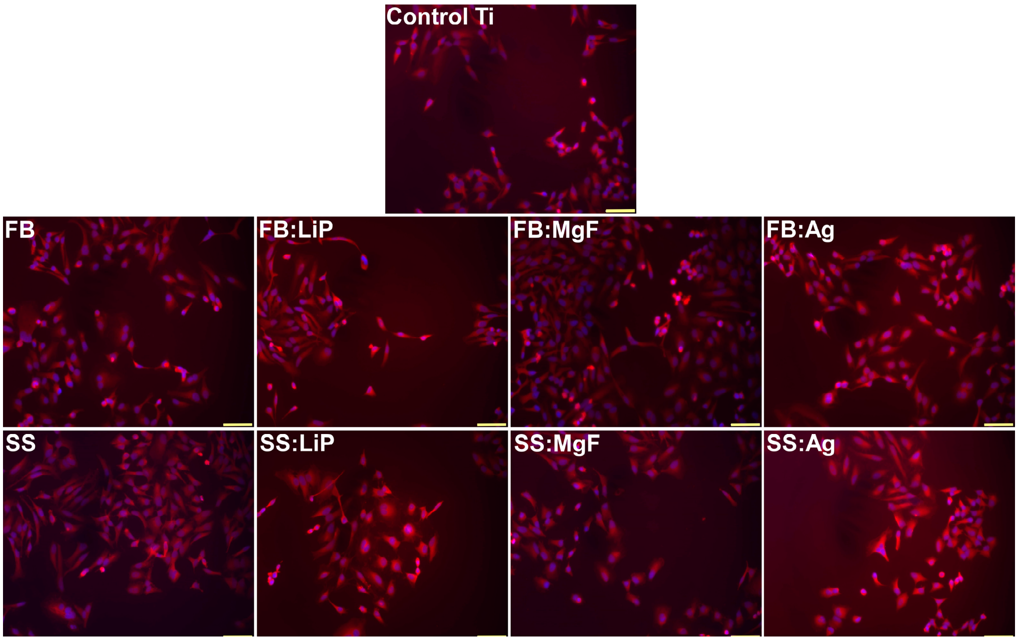

Figure S4. Fluorescence microscopy images acquired for control Ti and simple and doped FB and SS thin films using epithelial cells (HeLa). Magnification bar: 100 µm.

3. Antimicrobial Activity

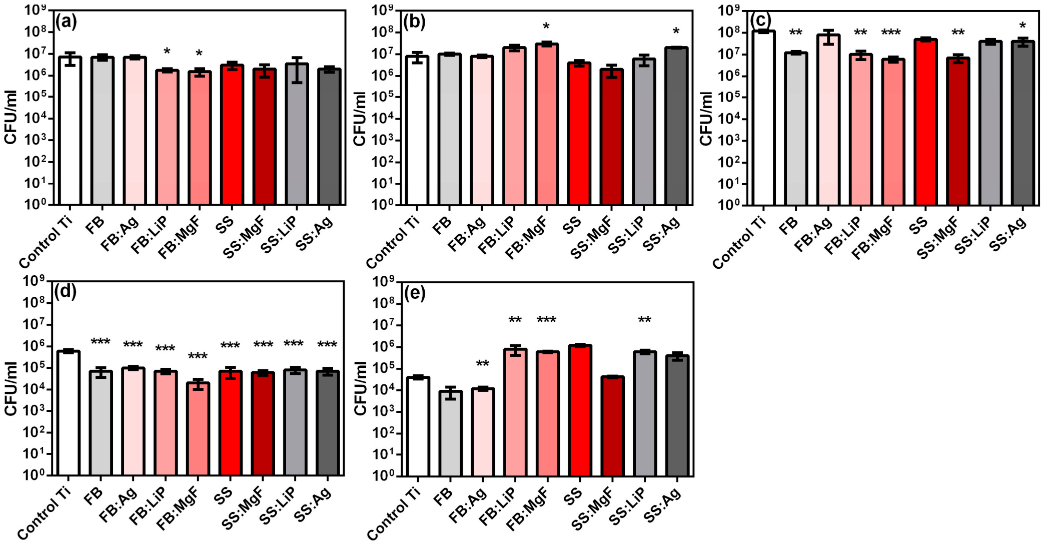

Figure S5. Number of microbial viable cells recovered from the biofilms developing on control Ti and simple and doped FB and SS thin films at T_0 (**a**), 2 h (**b**), 4 h (**c**), 24 h (**d**), and 48 h (**e**) using the *E. coli* strain (**p* < 0.05; ***p* < 0.01; ****p* < 0.0001).

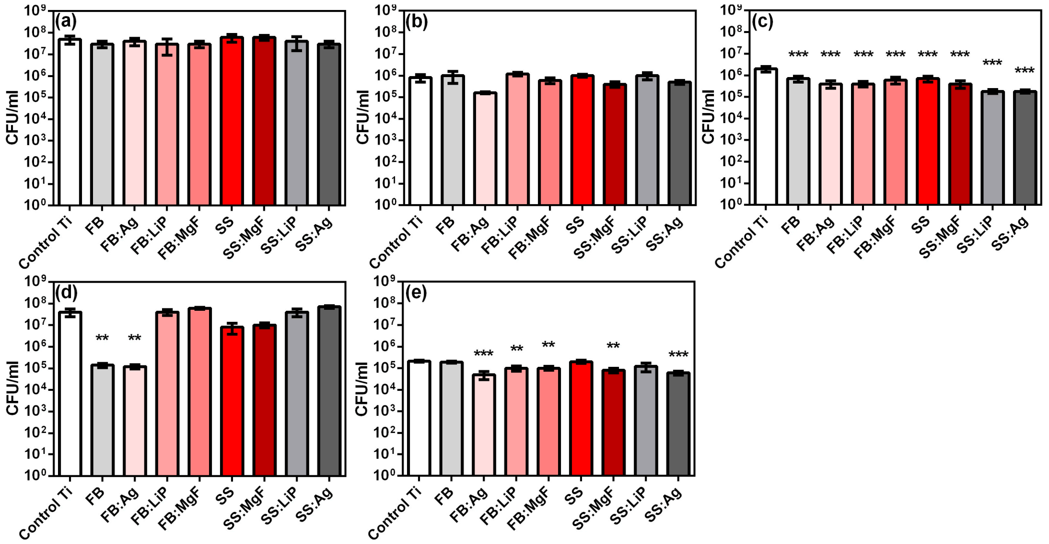

Figure S6. Number of microbial viable cells recovered from the biofilms developing on control Ti and simple and doped FB and SS thin films at T_0 (**a**), 2 h (**b**), 4 h (**c**), 24 h (**d**), and 48 h (**e**) using the *P. aeruginosa* strain (**p < 0.01; ***p < 0.0001).

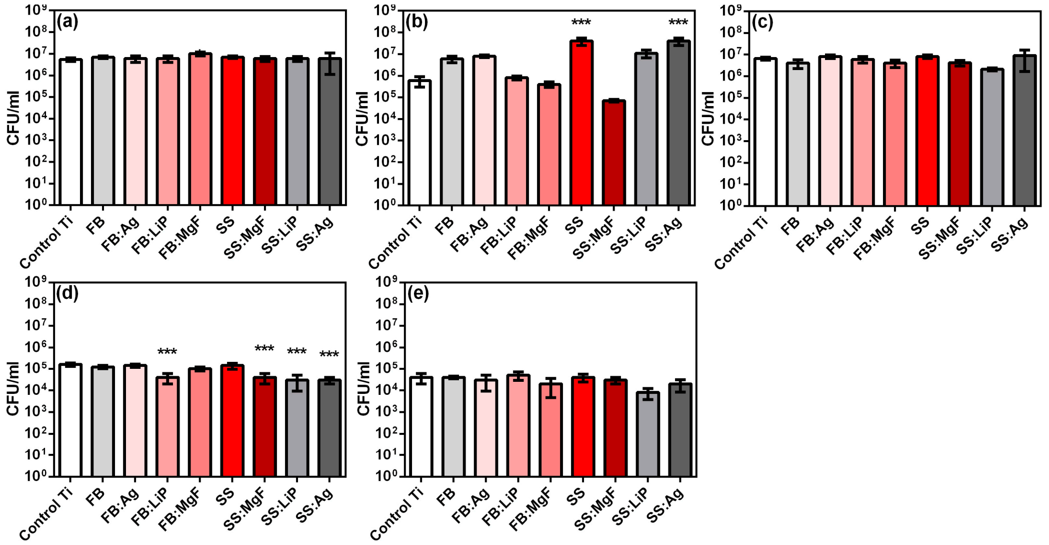

Figure S7. Number of microbial viable cells recovered from the biofilms developing on control Ti and simple and doped FB and SS thin films at T_0 (**a**), 2 h (**b**), 4 h (**c**), 24 h (**d**), and 48 h (**e**) using the *S. aureus* strain (***p < 0.0001).

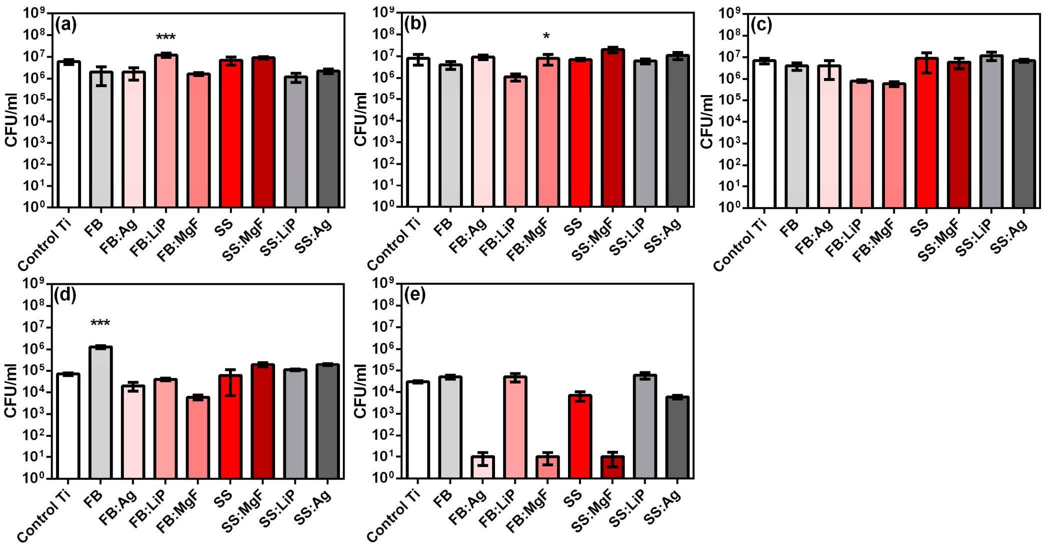

Figure S8. Number of microbial viable cells recovered from the biofilms developing on control Ti and simple and doped FB and SS thin films at T_0 (**a**), 2 h (**b**), 4 h (**c**), 24 h (**d**), and 48 h (**e**) using the *E. faecalis* strain (*$p < 0.05$; ***$p < 0.0001$).

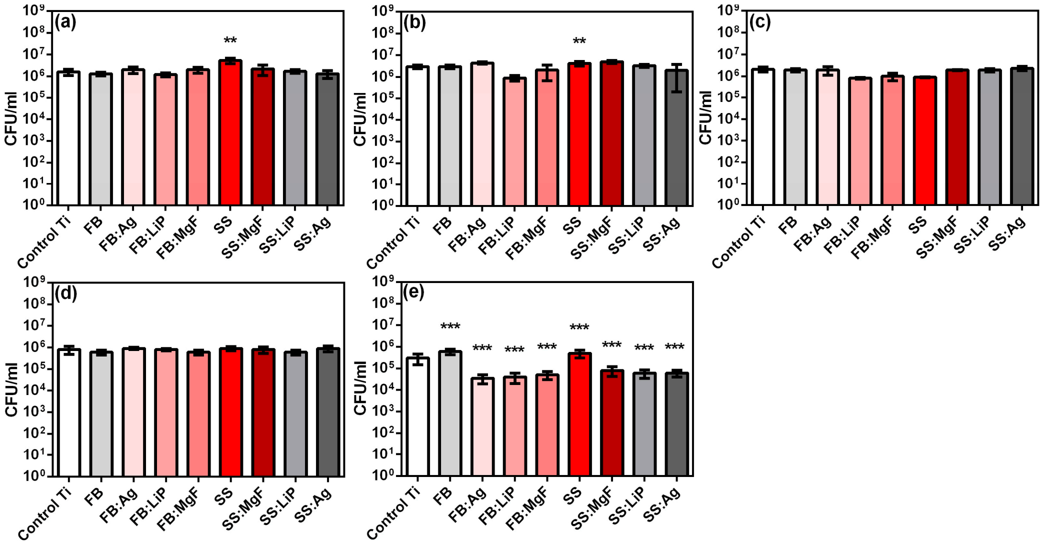

Figure S9. Number of microbial viable cells recovered from the biofilms developing on control Ti and simple and doped FB and SS thin films at T_0 (**a**), 2 h (**b**), 4 h (**c**), 24 h (**d**), and 48 h (**e**) using the *C. albicans* strain (**$p < 0.01$; ***$p < 0.0001$).
